# Identification and validation of CDC20 and ITCH as ubiquitination related biomarker in idiopathic pulmonary fibrosis

**DOI:** 10.1186/s41065-025-00401-y

**Published:** 2025-04-01

**Authors:** Shulei Sun, Yubao Wang, Jing Feng

**Affiliations:** Department of Respiratory and Critical Care Medicine, Tianjin Medical University General Hospital, 154 Anshan Road, Heping District, Tianjin, 300052 China

**Keywords:** Ubiquitination related genes, IPF, Bioinformatics analysis, Gene expression omnibus dataset, CDC20, ITCH

## Abstract

**Purpose:**

Ubiquitination plays a crucial role in various diseases. This study aims to explore the potential ubiquitination related genes in IPF.

**Methods:**

The gene microarray dataset GSE24206 was obtained from GEO database. Subsequently, through differential expression analysis and molecular signatures database, we obtained 1734 differentially expressed genes and 742 ubiquitination related genes. Through the venn diagram analysis, we obtained 53 differentially expressed ubiquitination related genes. Then, gene-ontology (GO) enrichment analysis, Kyoto Encyclopedia of Genes and Genomes (KEGG) pathway enrichment analysis, protein-protein interactions (PPI) and gene set enrichment analysis (GSEA) were applied for the differentially expressed ubiquitination related genes. Finally, the expression of CDC20 and ITCH in IPF patients and cells were validated by qPCR and western blot assay.

**Results:**

A total of 53 differentially expressed ubiquitination related genes (36 up-regulated genes and 17 down-regulated genes) were identified between 17 IPF patients and 6 healthy controls. GO and KEGG enrichment analysis of ubiquitination related genes mainly involved in regulation of protein ubiquitination, regulation of post-translational protein modification and ubiquitin mediated proteolysis. The PPI results demonstrated that these ubiquitination related genes interacted with each other. The GSEA analysis results for some of the hub genes mainly involved epithelial mesenchymal transition, inflammatory response, hypoxia, and apoptosis. The experiment expression level of CDC20 and ITCH in IPF patients and IPF cells were consistent with the bioinformatics analysis results.

**Conclusion:**

We identified 53 potential ubiquitination related genes of IPF through bioinformatics analysis. CDC20 and ITCH and other ubiquitination related genes may influence the development of IPF through epithelial mesenchymal transition and inflammatory response. Our research findings provide insights into the mechanisms of fibrosis and may provide evidence for potential therapeutic targets for fibrosis.

**Supplementary Information:**

The online version contains supplementary material available at 10.1186/s41065-025-00401-y.

## Introduction

Idiopathic pulmonary fibrosis (IPF) is chronic interstitial lung diseases. Its characteristic is the abnormal fibrosis and remodeling of tissues in the distal lung due to changes in cellular composition [1, 2]. The cause of idiopathic pulmonary fibrosis (IPF) is unclear. However, alveolar epithelial cell injury, activation of myofibroblast, and extracellular matrix deposition are important risk factors of IPF [3–5]. The currently available treatment drugs, such as the antifibrotic drugs pirfenidone and nintedanib, do not halt the progression of the disease over time [6–8]. In order to control the development of IPF, it is necessary for us to have a better understanding of the pathological process of IPF.

Ubiquitination is a post-translational modification of proteins that plays a crucial role in various diseases, involving at least three types of enzymes, including ubiquitin activating enzyme (E1), ubiquitin binding enzyme (E2), and ubiquitin ligase (E3) [9, 10]. Ubiquitination plays a critical role in the development and progression of various diseases. For instance, USP7 is a key deubiquitinase that regulates RAS stability, and targeting USP7 is a promising strategy for combating KRAS inhibitor resistance in non-small cell lung cancer (NSCLC) [11]. Another study suggests that SYVN1 suppresses ER stress through the ubiquitination and degradation of SIRT2 to block EMT process, thereby protecting against airway remodeling in asthma [12]. However, there are relatively few studies on the relationship between IPF and ubiquitination.

Meltzer EB et al. completed one IPF related dataset GSE24206, which analyzed gene microarray data between IPF patients and healthy individuals [13]. In this study, our aim is to explore ubiquitination related genes of IPF. We analyzed the differentially expressed genes of lung tissues from IPF patients and healthy lung tissues in the GEE24202 dataset. In addition, ubiquitination related genes were obtained through venn diagram analysis. Additionally, Gene Ontology (GO) and the Kyoto Encyclopedia of Genes and Genomes (KEGG) and protein-protein interactions (PPI) and gene set enrichment analysis (GSEA) were used to analyze ubiquitination related genes. Finally, the expression of CDC20 and ITCH in IPF patients and cells were validated by qPCR and western blot assay. By analyzing the ubiquitination related genes of IPF, we hope to enhance our understanding of the development of IPF and provide new insights into its diagnosis and treatment.

## Materials and methods

### Ubiquitination related genes database and gene microarray data

A total of 742 genes were obtained from the molecular signatures database (https://www.gsea-msigdb.org/gsea/msigdb/index.jsp). We downloaded one gene expression dataset with the number GSE24206 from the GEO database (http://www.ncbi.nlm.nih.gov/geo/). GSE24206 contained 17 IPF patients and 6 normal lung tissues, which were performed on the GPL570 platform.

### Differentially expressed analysis of ubiquitination related genes

The samples of GSE24206 dataset include IPF group and normal control group. The differences between the two groups were analyzed through principal component analysis (PCA) analysis using R software. Differentially expressed genes (DEGs) between IPF group and control group were analyzed using “limma” in R software. For GSE24206 gene expression profiles, *P* values < 0.05 and absolute fold-change value > 1.5 were selected as the cutoff values. The volcano plot, heatmap, and box plot of differentially expressed genes conducted using using the “heatmap” and “ggplot2” packages in R software. Ubiquitination differentially expressed genes were obtained through venn diagram analysis.

### GO and KEGG

Differential expression related genes in IPF were used for Gene Ontology (GO) and the Kyoto Encyclopedia of Genes and Genomes (KEGG) pathway enrichment analysis. All calculations were performed through the “clusterProfilter” package in R software. GO enrichment mainly includes biological processes (BP), molecular functions (MF), and cellular components (CC).

### PPI and correlation

Protein-protein interactions network of differentially expressed genes was conducted through STRING database (https://string-db.org/). An association was considered significant if the combined score was > 0.4. In addition, the network between proteins was presented through Cytoscape software (version 3.8.1). The expression correlation of differentially expressed genes was performed using the “corrplot” package in R software.

### Transcription factors (TF) of hub genes

The potential transcription factors of hub genes were predicted using the NetworkAnalyst database (https://www.networkanalyst.ca/). The relevant data of transcription factors were obtained from the JASPAR database (https://jaspar.elixir.no/). The results of transcription factor prediction were presented using Cytoscape software.

### GSEA

GSEA is used to evaluate the distribution trend of genes from a predefined gene set in the gene list ranked with phenotypic relevance, to judge its contribution to phenotype. The expression results calculated based on the “limma” package were used for GSEA. GSEA was conducted using the gene sets from “Hallmark gene sets”, utilizing the GSEA method from the R package “clusterProfiler”. For single-gene GSEA, the samples were divided into high-expression groups and low-expression groups according to the expression levels of TP53, MYC, CDK1, ITCH, NFKBIA and CDC20. The differentially expressed genes between the two groups were calculated using the limma package of R software.

### Experimental validation of expression of ITCH and CDC20 gene

A total of 6 IPF patients and 6 age-matched healthy individuals were obtained from the Tianjin Medical University General Hospital between May 2022 and January 2024. This study was performed in accordance with the Declaration of Helsinki and approved by the Medical Ethics Committee of the hospital. Peripheral blood was collected from all participants. Total RNA was extracted using the RNA extraction kit from peripheral blood mononuclear cells (PBMCs). The reverse transcription and qPCR procedures were conducted following the manufacturer’s instructions (Takara, Dalian, China). The primer sequences of the study were shown in Table 1. ACTB was used as the internal reference gene.

**Table 1:** Primer sequences used for qPCR

Primer	5’-3’
*ITCH*-qPCR-F *ITCH*-qPCR-R *CDC20*-qPCR-F *CDC20*-qPCR-R *ACTB*-qPCR-F *ACTB*-qPCR-R	CTCTCTGCCGCCGACAAATA TGGCAAGGGAGCTTGAGTTAC GCACAGTTCGCGTTCGAGA CTGGATTTGCCAGGAGTTCGG TGGCACCCAGCACAATGAA CTAAGTCATAGTCCGCCTAGAAGCA

### MRC-5 cells culture and treatment

MRC-5 cells were purchased from ATCC and cultured in MEM medium supplemented with 10% fetal bovine serum and incubated at 37 °C with 5% CO_2_. For the IPF cell model, MRC-5 cells need to be serum starved for 24 h and then treated with TGF-β1 (5 ng/mL) for 24 h.

### Western blot

Cell protein extraction was performed using RIPA lysis buffer with added protease inhibitors (Beyotime, China). The protein samples were separated using SDS-PAGE. The protein samples were subsequently transferred to the polyvinylidene fluoride (PVDF) membranes and blocked with a 5% skimmed milk solution. Then, the membranes incubated with primary antibody and secondary antibody. The antibodies used included anti-ITCH (Proteintech, China), anti-CDC20 (Proteintech, China), anti-α-SMA (Proteintech, China), and anti-β-ACTIN (Proteintech, China).

### Statistical analysis

The statistics analyses were performed using R software (version 3.6.2) and Graphpad Prism (version 6.0). *P* < 0.05 was considered statistically significant.

## Results

### Identification of DEGs between IPF and normal groups

**Fig. 1:**
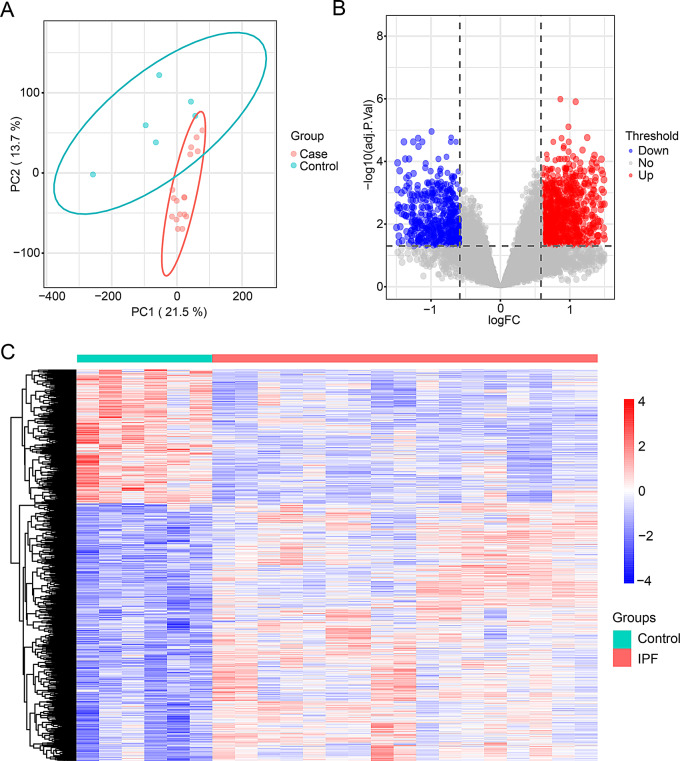
Differentially expressed genes between the idiopathic pulmonary fibrosis and normal groups. (**A**) Principal component analysis of the IPF and normal samples. (**B**) Volcano plot analysis of differentially expressed genes between the IPF and normal groups. (**C**) Heatmap analysis of differentially expressed genes between the IPF and normal groups

To evaluate the reproducibility of the data within the two groups, we performed principal component analysis (PCA). The results showed that there were differences between the two groups (Fig. 1A). By conducting differential expression genes in 17 IPF lung samples and 6 normal lung samples, we found 1734 differentially expressed genes, including 1140 up-regulated genes and 594 down-regulated genes. The differential expression genes ware visualized by volcano plot and heatmap, as shown in Fig. 1B and C.

### GSEA analysis between IPF and normal groups

**Fig. 2:**
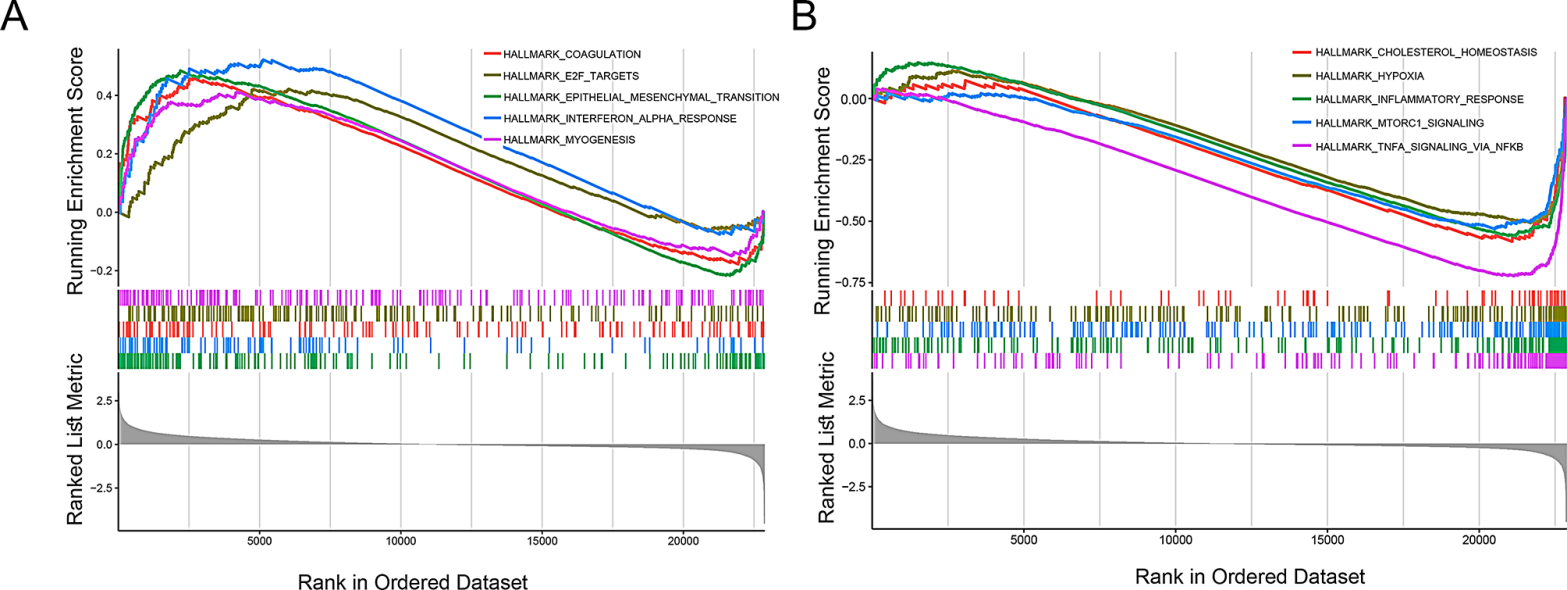
GSEA results of differentially expressed genes in idiopathic pulmonary fibrosis. (**A**) Top five positive enrichment results in the IPF group compared to the normal group. (**B**) Top five negative enrichment results in the IPF group compared to the normal group

We performed GSEA analysis on the IPF and normal groups. The results indicated that the positive enriched terms were primarily related to epithelial mesenchymal transition and interferon alpha response, while the negative enriched terms were mainly associated with inflammatory response, hypoxia, and cholesterol homeostasis (Fig. 2A and B).

### Identification of ubiquitination related genes in IPF

**Fig. 3:**
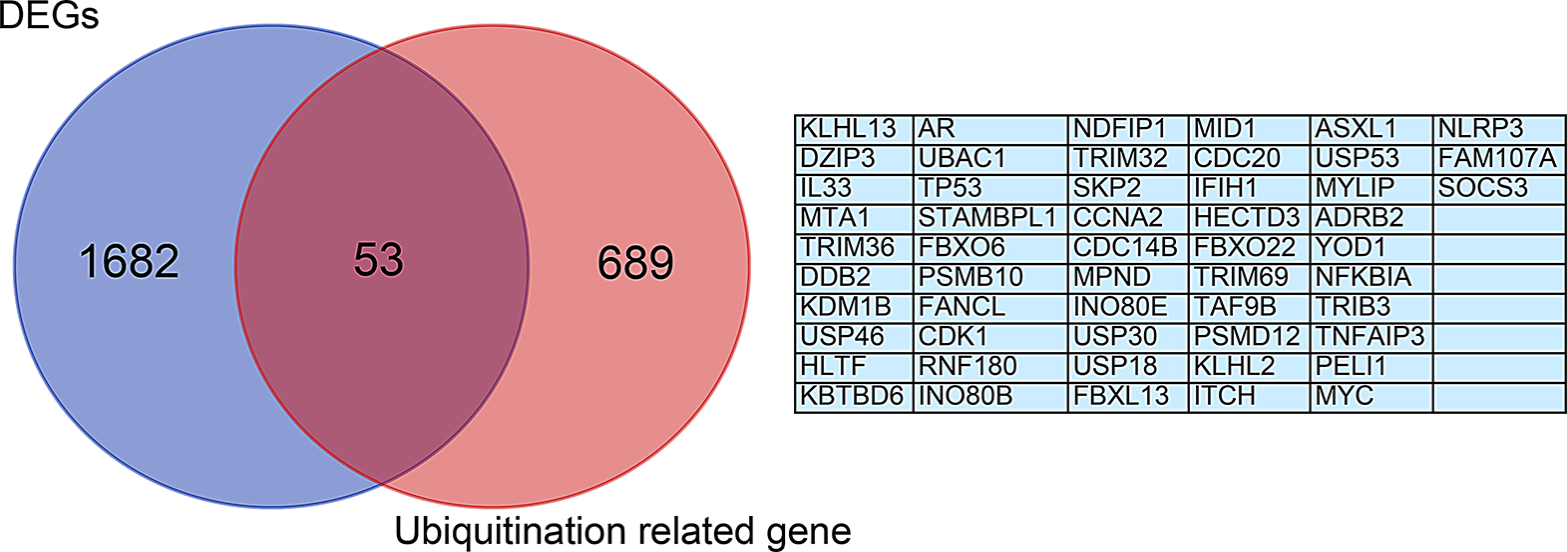
Venn diagram analysis of differentially expressed genes and ubiquitination related genes

To explore the ubiquitination related genes of IPF, we obtained 742 ubiquitination related genes from the molecular signatures database (MSigDB). Through venn diagram analysis, 53 genes were identified as overlapping genes, which are referred to as ubiquitination related genes (Fig. 3).

### The expression of ubiquitination related genes in IPF

**Fig. 4:**
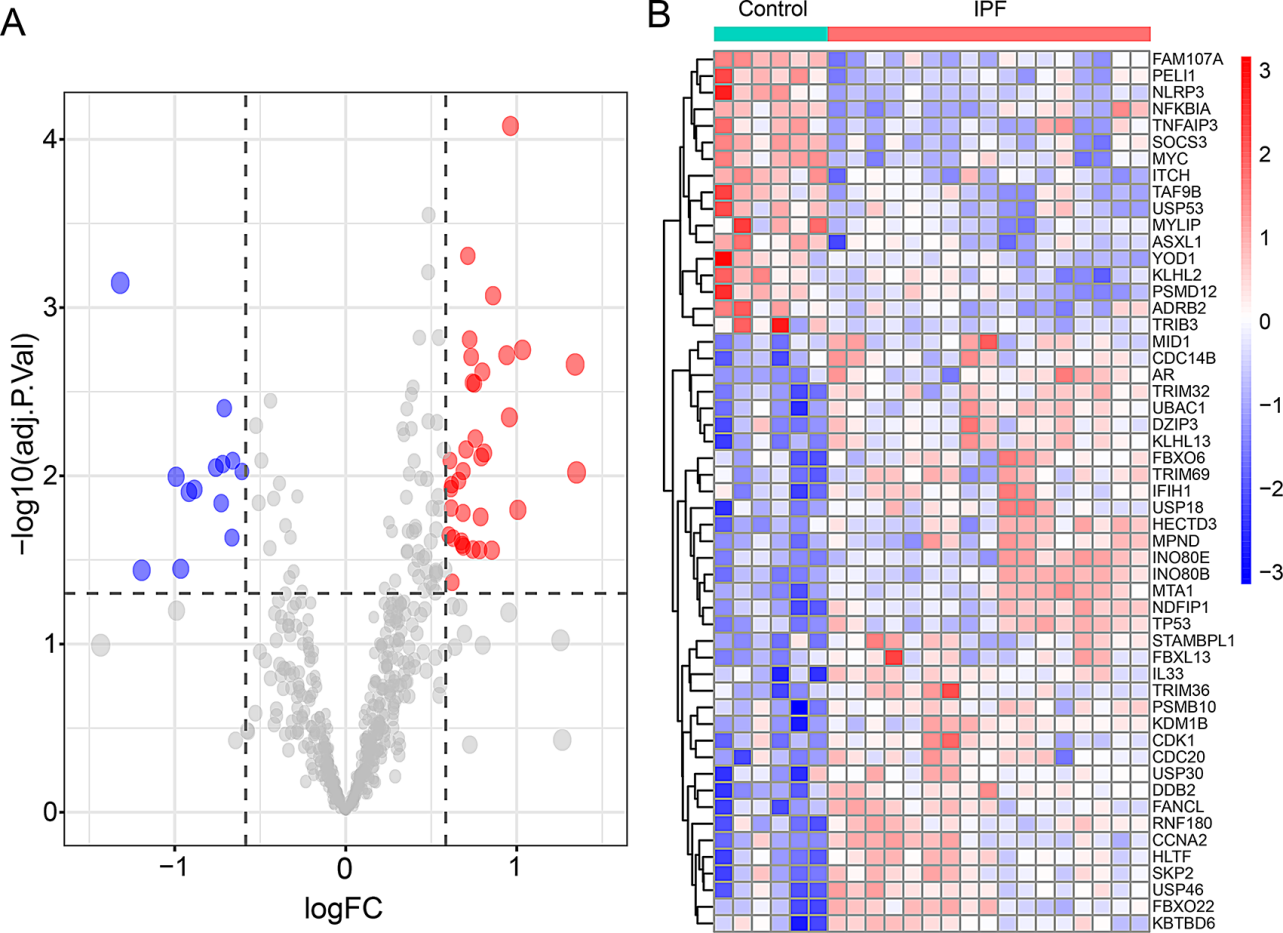
Differentially expressed ubiquitination related genes between the IPF group and normal group. (**A**) Volcano plot analysis. (**B**) Heatmap analysis

**Fig. 5:**
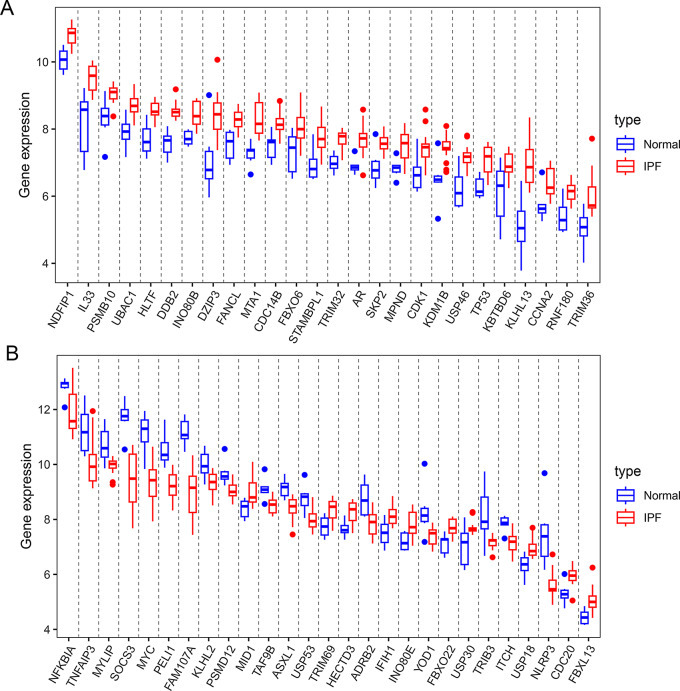
The boxplot of 53 differentially expressed ubiquitination related genes in IPF and healthy samples. (**A**) The boxplot of top 26 differentially expressed ubiquitination related genes in IPF and healthy samples. (**B**) The boxplot of last 27 differentially expressed ubiquitination related genes in IPF and healthy samples

We further analyzed 53 ubiquitination related genes in IPF, and the results of volcano plots and heatmap indicated 36 up-regulated genes and 17 down-regulated genes (Fig. 4A and B). In addition, box plots showed the expression of 53 ubiquitination related genes between IPF and normal samples (Fig. 5A and B).

### GO and KEGG enrichment analysis of ubiquitination related genes

**Fig. 6:**
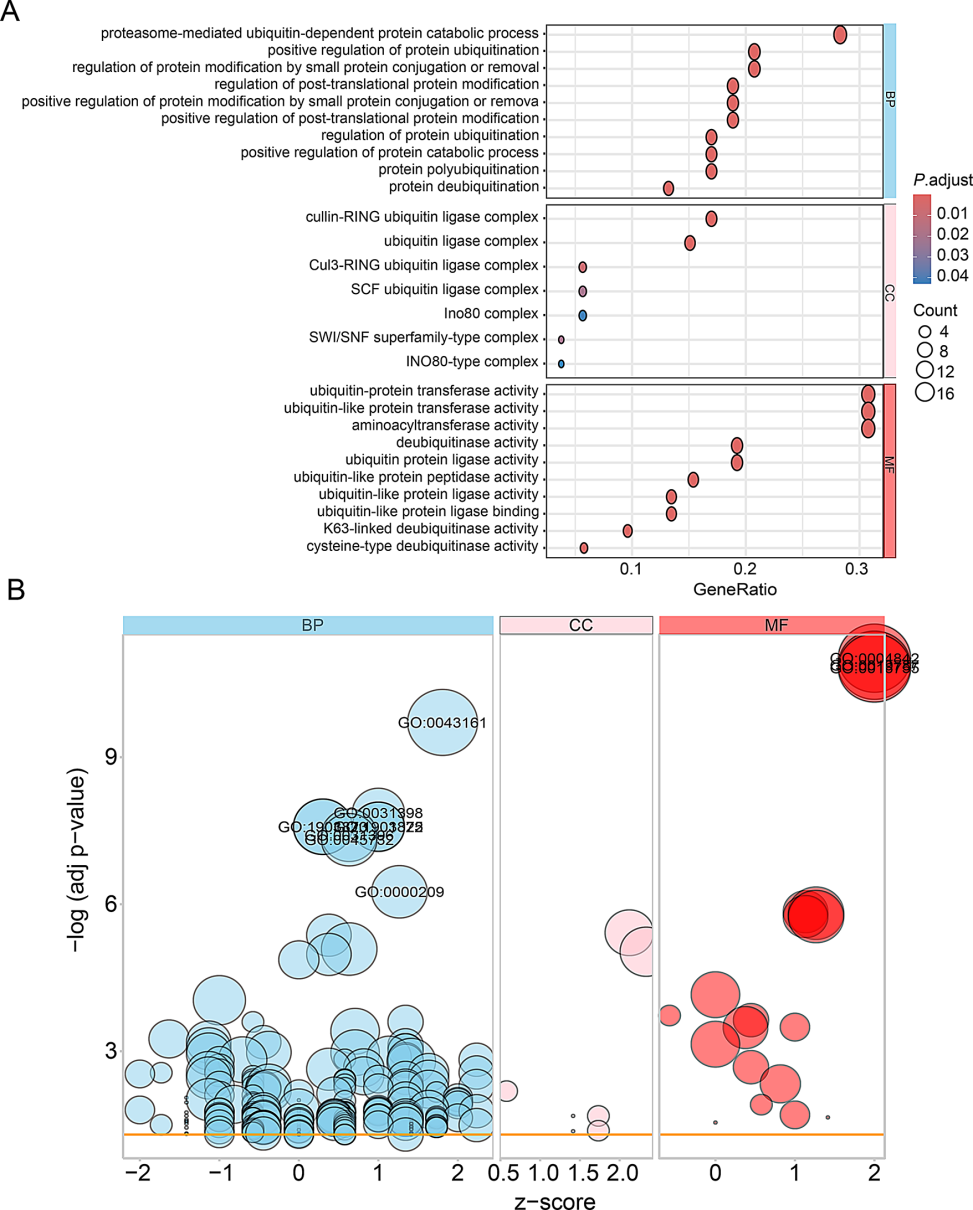
Gene Ontology (GO) enrichment analysis of 53 differentially expressed ubiquitination related genes. (**A**) and (**B**) Bubble plot of enriched GO terms. Abbreviations: BP, biological process; CC, cellular component; MF, molecular function

To analyze the potential biological functions of these ubiquitination related genes, GO and KEGG enrichment analysis were performed. Predominant BP pathways were pertinent to proteasome-mediated ubiquitin-dependent protein catabolic process, regulation of protein ubiquitination and regulation of post-translational protein modification. Examination of CC revealed a remarkable enrichment of cullin-RING ubiquitin ligase complex, ubiquitin ligase complex and SCF ubiquitin ligase complex. In terms of MF, activities related to the ubiquitin-protein transferase activity, ubiquitin-like protein transferase activity and ubi quitin-like protein ligase activity (Fig. 6A and B; Supplementary Table S1).

**Fig. 7:**
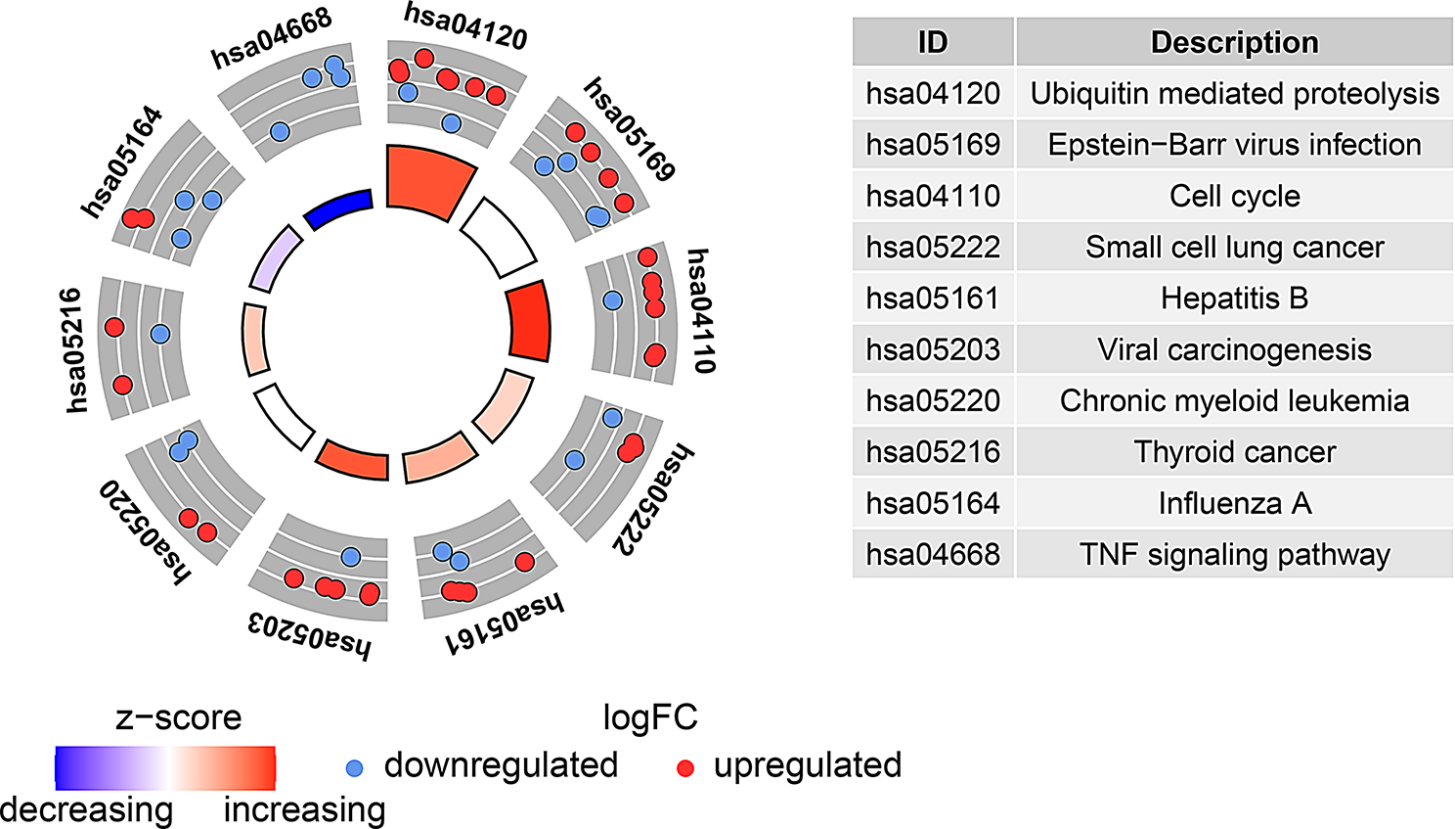
Kyoto Encyclopedia of Genes and Genomes (KEGG) analysis of 53 differentially expressed ubiquitination related genes

Additionally, according to KEGG analysis, the ubiquitination related genes mainly involved in the ubiquitin mediated proteolysis, cell cycle and TNF signaling pathway (Fig. 7; Supplementary Table S2).

### Establishment of PPI network and identification of hub genes

**Fig. 8:**
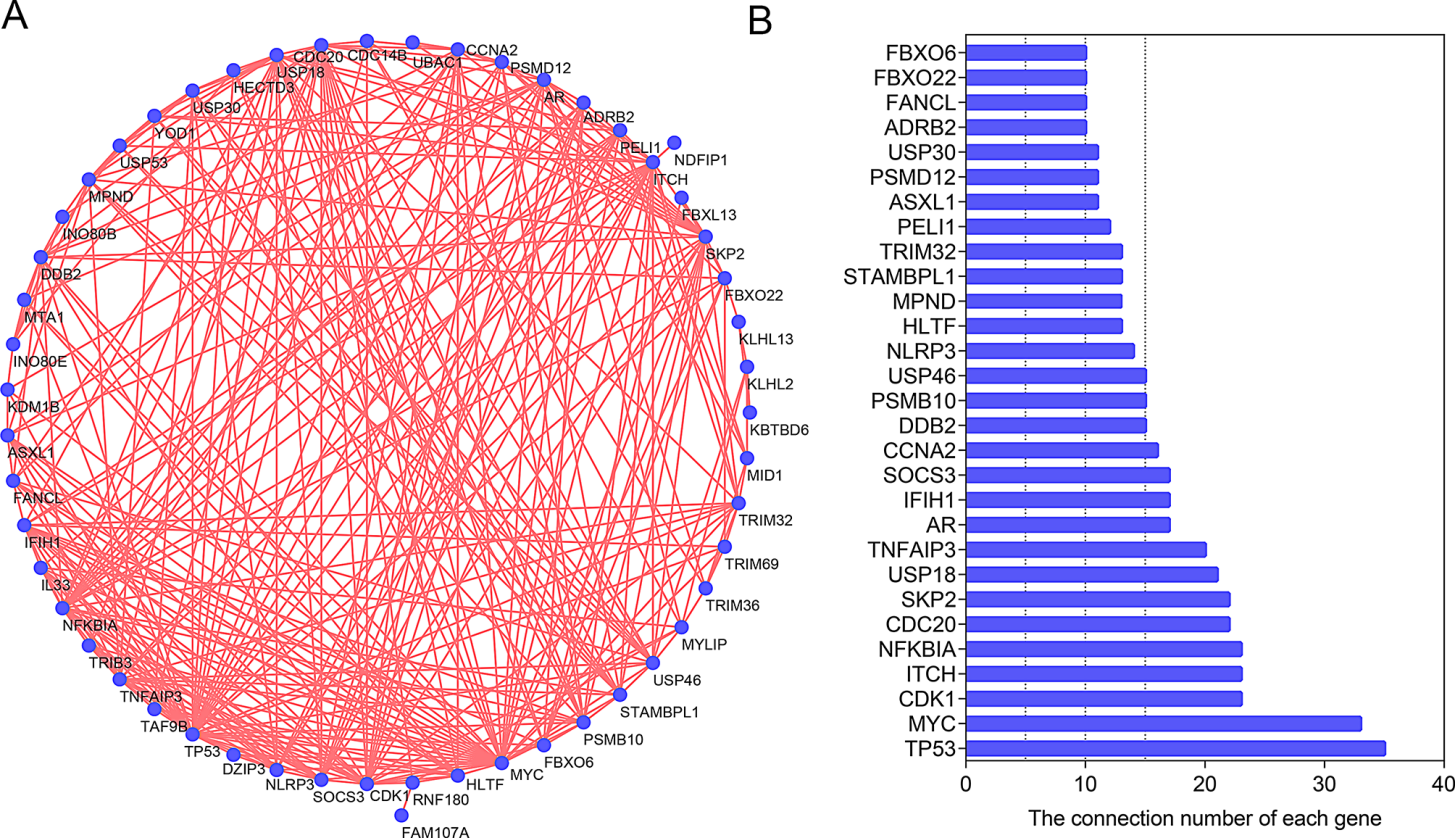
Protein-protein interactions (PPI) analysis 53 differentially expressed ubiquitination related genes. (**A**) The PPI among ubiquitination related genes. (**B**) The interaction number of each differentially expressed ubiquitination related genes

**Fig. 9:**
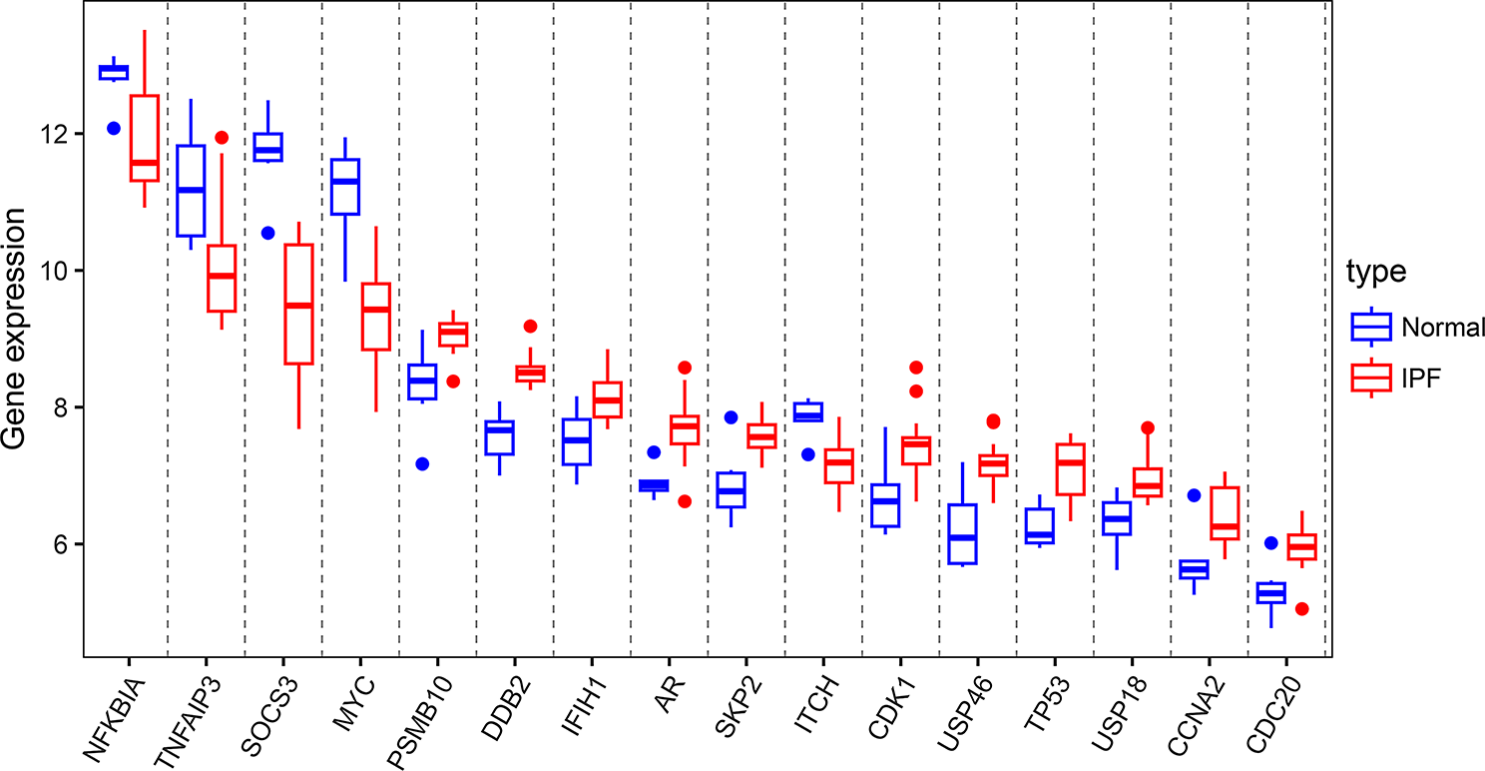
The boxplot of 16 hub ubiquitination related genes in IPF and healthy samples

To assess the interactions between differentially expressed ubiquitination related genes, we conducted PPI network analysis. The analysis revealed that these ubiquitination related genes formed an interaction network and showed the interaction count for each gene (Fig. 8A and B). Based on the results of the PPI network analysis, genes with more than 15 interactions were defined as hub genes. The box plots showed the expression of 16 hub genes (Fig. 9).

### Correlation analysis and transcription factors (TF) target gene network construction of hub genes

**Fig. 10:**
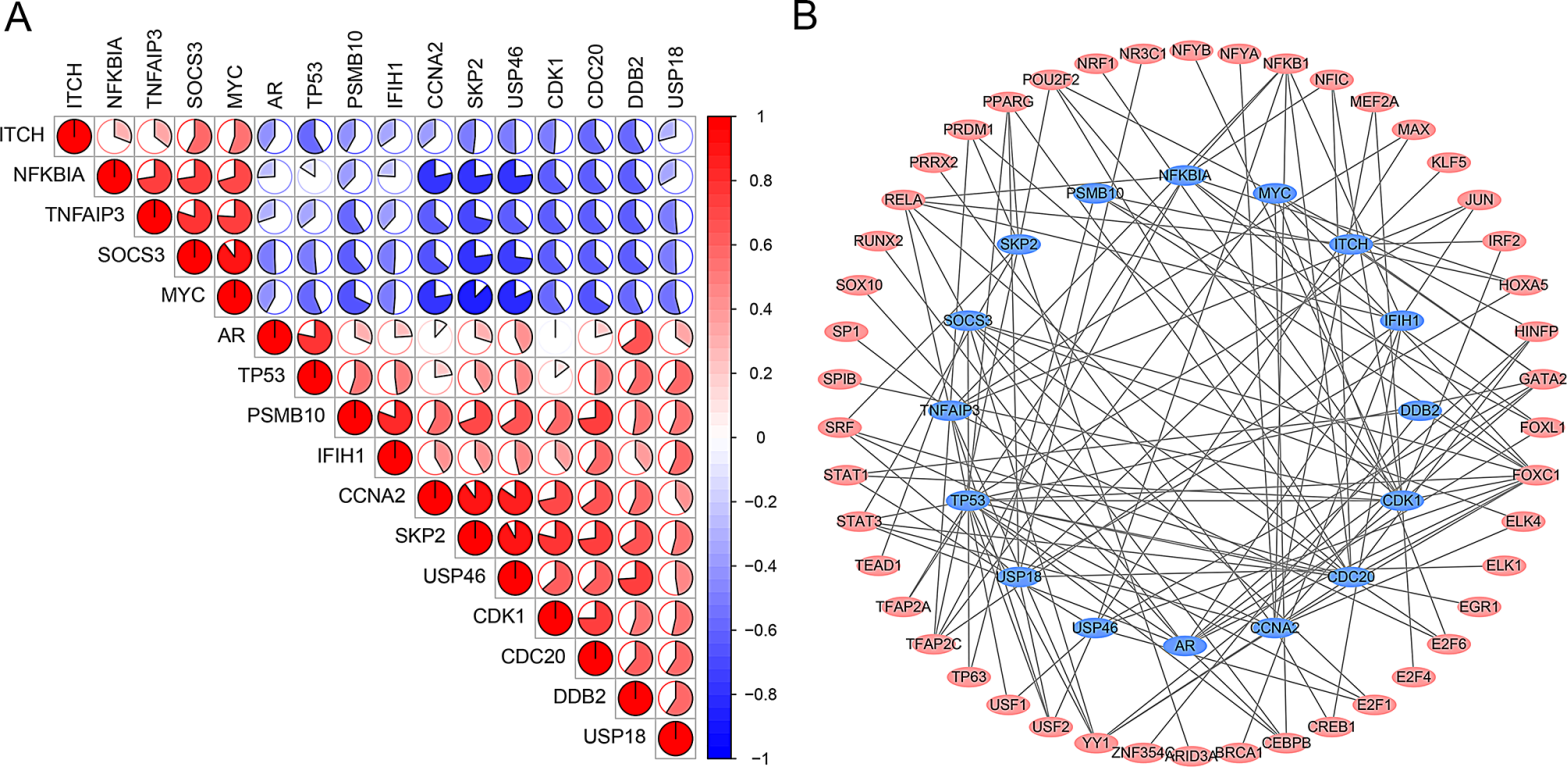
Correlation analysis and transcription factors target gene network construction of hub genes. (**A**) Correlation analysis of the 16 differentially expressed ubiquitination related genes. (**B**) Transcription factors-hub genes interaction network

To explore the expression correlation of the 16 hub genes, correlation analysis was performed. The results showed the relationship of these genes in Fig. 10A. To predict the transcription factors that interact with the 16 hub genes, we utilized NetworkAnalyst database, and the resulting transcription factor-gene regulatory network was visualized using Cytoscape (Fig. 10B).

### Single-gene GSEA results of hub genes

**Fig. 11:**
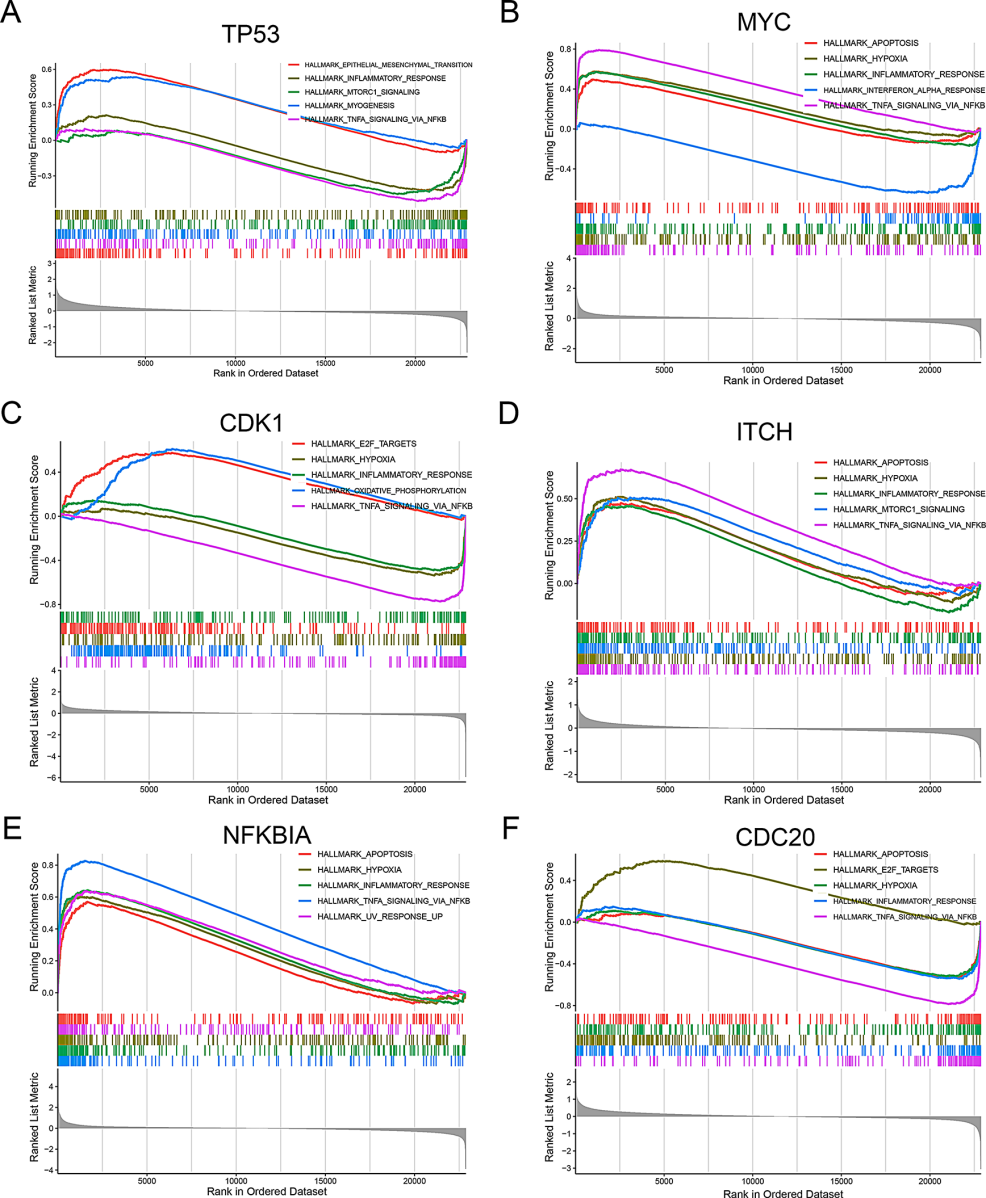
GSEA enrichment results of six hub genes. (**A**) TP53. (**B**) MYC. (**C**) CDK1. (**D**) ITCH. (**E**) NFKBIA. (**F**) CDC20

To better understand the function of the hub genes in IPF, we divided the samples into high-expression and low-expression groups using the median gene expression value as the threshold, and then performed single-gene GSEA analysis on these two groups. The results revealed that these genes participated in epithelial mesenchymal transition, inflammatory response, hypoxia and apoptosis (Fig. 11A-F).

### The experimental validation of *CDC20* and *ITCH* in IPF patients and cells

**Fig. 12:**
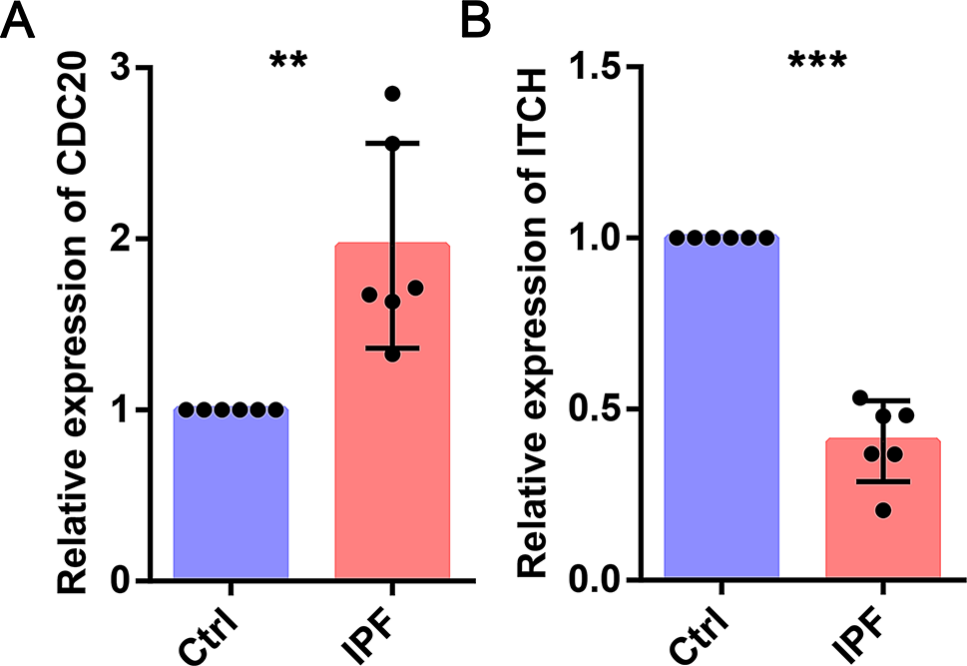
mRNA expression of CDC20 and ITCH genes were measured in IPF patients and healthy samples. (**A**) CDC20. (**B**) ITCH

**Fig. 13:**
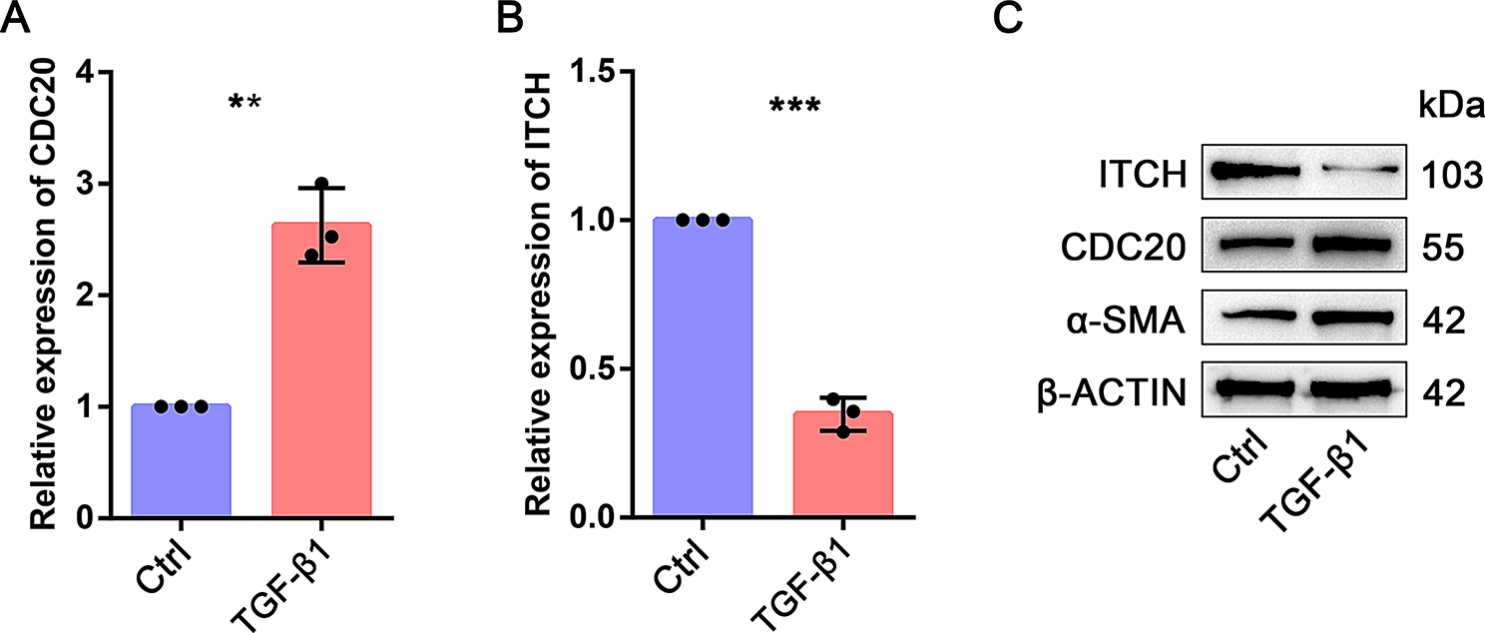
RNA and protein expression of CDC20 and ITCH genes were measured in IPF patients and healthy samples. (**A**-**B**) qPCR assay was used to detect the mRNA expression of CDC20 and ITCH. (**C**) Western blot assay was used to detect the protein expression of CDC20, ITCH and α-SMA

According to our database search, some hub genes have been explored in IPF. However, CDC20 and ITCH have not been reported in IPF, so we chose these two genes for further exploration. qPCR was used to detect the mRNA expression of *CDC20* and *ITCH* of IPF patients. The expression level of *CDC20* was significantly higher in IPF patients than in control group (Fig. 12A). In addition, the expression level of *ITCH* was significantly decreased (Fig. 12B). We then identified CDC20 and ITCH expression in MRC-5 cells treated with TGF-β1. The mRNA and protein expression levels of CDC20 were markedly upregulated in TGF-β1 group compared with control group (Fig. 13A and C). However, the mRNA and protein expression of ITCH was significantly decreased in TGF-β1 group (Fig. 13B and C). Our experiment results are consistent with the results of bioinformatics.

## Discussion

Ubiquitination is a post-translational modification where ubiquitin, a small regulatory protein, is attached to substrate proteins [14]. This process targets proteins for degradation via the proteasome, influences protein function, localization, and interactions, playing critical roles in various cellular processes including cell cycle control and DNA repair [15]. Ubiquitination related genes and proteins play an important role in clinical drug development, especially in fields such as tumors and neurodegenerative diseases. For example, Bortezomib and Carfilzomib are approved proteasome inhibitors used in the treatment of multiple myeloma [16, 17]. In addition, drug development targeting specific ubiquitin E3 ligases has attracted much attention, such as the small molecule inhibitor Nutlin targeting MDM2, which can block the degradation of p53 and enhance its anti-tumor activity [18, 19]. Our results identified some ubiquitination related genes in IPF, which may provide theoretical basis for the development of potential therapeutic targets for IPF.

The course of IPF is marked by a poor prognosis and unpredictability, leading to irreversible advancement and eventually culminating in respiratory failure and fatal outcomes [20]. Despite numerous studies published in the field, the exact mechanisms of IPF onset and progression remain largely unknown. It is necessary to explore the pathogenesis of IPF, and many scholars have focused on the epigenetic aspects of IPF [21]. Epigenetics includes aspects such as DNA methylation and protein ubiquitination and phosphorylation. These epigenetic modifications play an important role in the development of diseases. Zhang et al. found that YTHDC1 delays cellular senescence and pulmonary fibrosis by activating ATR in an m6A methylation-independent manner [22]. Another study suggested that inhibition of ROCK ameliorates pulmonary fibrosis by suppressing M2 macrophage polarization through the phosphorylation of STAT3 [23]. These findings indicate that epigenetics can influence the development of IPF.

Several articles utilize CEO databases to analyze the potential molecular mechanisms of IPF from different perspectives, such as oxidative stress and mitochondria related genes [24, 25]. However, there have been no reports on the association between ubiquitination related genes and IPF. Based on the Venn diagram analysis in Fig. 3, we identified 53 potential ubiquitination related genes of IPF through bioinformatics analysis. The volcano and heatmap in Fig. 4 show the expression of 53 differentially expressed ubiquitination related genes. GO and KEGG enrichment analysis were performed to investigate the potential biological functions of these ubiquitination related genes (Figs. 6 and 7). The enrichment results were mainly focused on protein ubiquitination and regulation of post-translational protein modification. In recent years, some studies have reported that ubiquitination is involved in the development of IPF. Recently, Yan et al. found that DNA-PKcs/AKT1 inhibits epithelial mesenchymal transition during radiation-induced pulmonary fibrosis by inducing ubiquitination and degradation of Twist1 [26]. Another study suggests that the ubiquitination pathway promoted by NEDD4 slows down the progression of IPF by downregulating the transcription of YY1 and TAB1 [27].

Through PPI analysis, we found that these ubiquitination related genes interact with each other (Fig. 8A). Figure 8B showed the detailed number of interactions between different proteins. According to the results in Fig. 8B, genes with more than 15 interactions were defined as hub genes. Hub genes can affect the pathophysiology of other diseases through ubiquitination. Chen et al. found that USP54 can ubiquitinate and degrade p53 expression, thereby affecting GLUT1-mediated aerobic glycolysis in lung adenocarcinoma [28]. Another study found that inhibiting Skp2 can promote ferroptosis by enhancing the ubiquitination of SLC3A2, thereby contributing to sepsis-induced acute lung injury [29]. Additionally, USP18 inhibits hepatic stellate cell activation and alleviates liver fibrosis by regulating TAK1 activity through ubiquitination [30]. In non-small cell lung cancer, Kremen2 drives tumor progression by preventing SOCS3-mediated ubiquitination of EGFR [31]. However, the investigation of these genes in IPF is still insufficient.

Furthermore, we also present the expression of the 16 hub genes using box plots in Fig. 9. We further analyzed the expression correlation of 16 hub genes in Fig. 10A. As shown in the results, the blue circle indicates a negative correlation between the expression of two genes, while the red circle indicates a positive correlation between the expression of two genes. We explored the potential upstream transcription factors of the hub genes and obtained regulatory relationship results of upstream transcription factors for some hub genes (Fig. 10B). These transcription factors may influence the occurrence and development of IPF by promoting or inhibiting the expression of the hub genes. Transcription factors have been reported to affect the pathophysiological processes of IPF. A study reported that GATA3 promotes the development of IPF by transcriptional activation of NRP1 expression [32]. Another study suggested that Sohlh2 can promote pulmonary fibrosis by transcriptional repression of the p62/Keap1/Nrf2 mediated anti-oxidative signaling pathway [33]. These transcription factors are also potential targets for IPF diagnosis and treatment. Ubiquitination-related enzymes primarily exert their effects by influencing the expression of downstream proteins. We plan to explore the potential downstream proteins of these ubiquitination enzymes in future studies.

We conducted single-gene GSEA analysis on six hub genes in Fig. 11, and the results mainly enriched the key biological processes and pathways of IPF, such as epithelial mesenchymal transition, inflammatory response, hypoxia, and apoptosis. Epithelial mesenchymal transition, inflammatory response, hypoxia and apoptosis are important risk factors for IPF [34–36]. Zhou et al. found that MiR-125b-5p alleviates pulmonary fibrosis by inhibiting the TGFβ1-mediated epithelial-mesenchymal transition through targeting BAK1 [37]. Another study suggests that Nintedanib mitigates radiation-induced pulmonary fibrosis by suppressing the inflammatory response in epithelial cells and inhibiting the transition of fibroblasts to myofibroblasts [38]. Additionally, hypoxia enhances the fibrogenic activity of IPF mesenchymal progenitor cells through the lactate/GPR81/HIF1α pathway [39]. It is necessary to explore the role of these hub genes in the development of IPF through epithelial mesenchymal transition and inflammatory response.

Based on other analysis results, we chose CDC20 and ITCH for further validation for the following reasons. First, CDC20 and ITCH are ranked among the top ten hub genes, indicating their significant roles in the regulatory network. In addition, while some of the highly ranked genes have already been explored in IPF, CDC20 and ITCH have not been investigated in IPF by other researchers. Through qPCR and WB experiments, we found that CDC20 is high expressed in IPF while ITCH is low expressed in IPF (Figs. 12 and 13). The results of the experiment are consistent with the analysis of bioinformatics. CDC20 is involved in the metaphase and anaphase transition of cell cycle [40]. CDC20 is necessary for full ubiquitin ligase activity of the anaphase promoting complex/cyclosome (APC/C) and confers substrate specificity upon the complex [41]. In addition, ITCH encodes a member of the Nedd4 family of HECT domain E3 ubiquitin ligases [42]. Recent research has indicated that ITCH mediates SIX1 ubiquitination and influences the development of nasopharyngeal carcinoma via the CDC27-cyclin B1 signaling pathway [43]. In summary, these findings indicate that CDC20 and ITCH can influence the pathological progression of various diseases through ubiquitination. However, these two proteins have not been reported in IPF, and we plan to explore their detailed mechanisms in IPF.

Multiple studies have reported that ubiquitination is involved in various respiratory diseases. For example, one study revealed that FBXL19 targets STK11 degradation to enhance cigarette smoke induced cytotoxicity in airway epithelial cells [44]. In addition, the deubiquitination of OAS3 due to the downregulation of the E3 ligase TRIM21 promotes epithelial cell apoptosis and drives sepsis-induced acute lung injury [45]. In lung adenocarcinoma, the deubiquitinated protein OTUD6B promotes tumor progression by stabilizing RIPK1 [46]. In asthma, LncTRPM2-AS inhibits TRIM21 mediated TRPM2 ubiquitination and prevents macrophage autophagy induced apoptosis [47]. In summary, ubiquitination plays a crucial role in various respiratory diseases.

Our research findings enhance our understanding of development of IPF from the perspective of ubiquitination modification. However, there are several limitations in our study. We analyzed the functional enrichment analysis and potential functional directions of ubiquitination related genes, but we did not conduct experimental verification. We plan to conduct further experimental verification in the future. In addition, the number of clinical samples analyzed in this study is not large enough, and we need to validate our results in a larger cohort in the future.

In summary, we identified 53 ubiquitination related genes in IPF through data analysis. The hub genes CDC20 and ITCH and other ubiquitination related genes may affect the development of IPF through epithelial mesenchymal transition and inflammatory response. Our research findings may provide potential targets for the diagnosis and treatment of IPF.

## Electronic supplementary material

Below is the link to the electronic supplementary material.

Supplementary Material 1

Supplementary Material 2

Supplementary Material 3

## Data Availability

Publicly available datasets were analyzed in this study. The datasets GSE24206 for this study can be found here: https://www.ncbi.nlm.nih.gov/geo/.

## Abbreviations

BP: Biological process
CC: Cellular component
GEO: Gene expression omnibus dataset
GO: Gene Ontology
KEGG: Kyoto Encyclopedia of Genes and Genomes
MF: Molecular function
PCA: Principal component analysis
PPI: Protein-protein interactions
GSEA: Gene set enrichment analysis
TF: Transcription factor
