# Supplementary figures and images for: Identification and validation of CDC20 and ITCH as ubiquitination related biomarker in idiopathic pulmonary fibrosis
Source: Hereditas. 2025 Apr 1;162:50. doi: 10.1186/s41065-025-00401-y (PMC11959808; doi:10.1186/s41065-025-00401-y)

## Slide 1
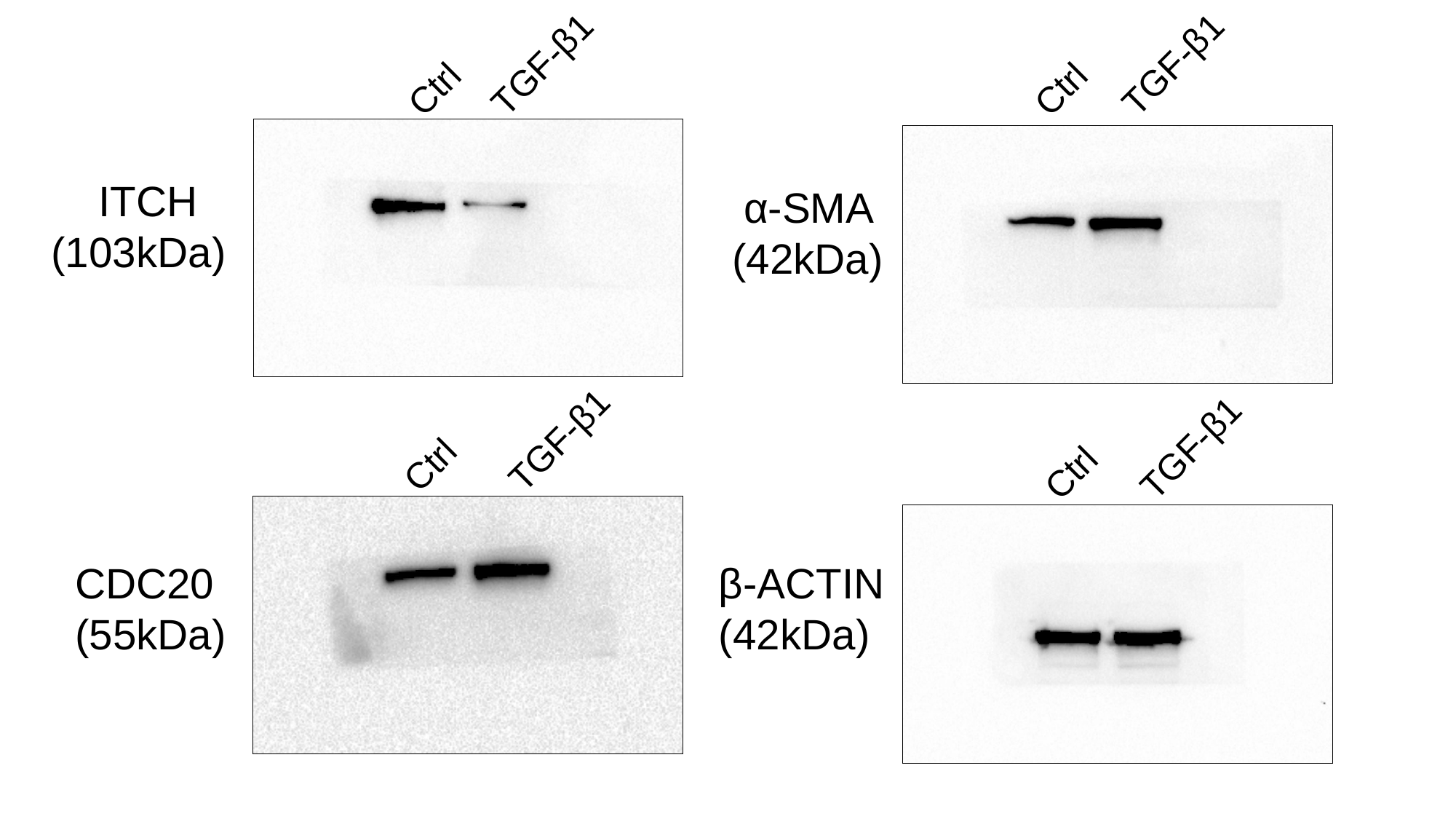

TGF-β1
TGF-β1
Ctrl
Ctrl
 ITCH
(103kDa)
 α-SMA
(42kDa)
TGF-β1
TGF-β1
Ctrl
Ctrl
CDC20
(55kDa)
β-ACTIN
(42kDa)

Supplement: Supplementary file 3 — Supplementary Material 3 [file 41065_2025_401_MOESM3_ESM.pptx]
